# Altered relationship between gluconeogenesis and immunity in broilers exposed to heat stress for different durations

**DOI:** 10.1016/j.psj.2021.101274

**Published:** 2021-05-21

**Authors:** Sharif Hasan Siddiqui, Darae Kang, Jinryong Park, Mousumee Khan, Shah Ahmed Belal, Donghyun Shin, Kwanseob Shim

**Affiliations:** ⁎ Department of Animal Biotechnology, Jeonbuk National University, Jeonju, Republic of Korea; † Department of Biomedical Sciences and Institute for Medical Science, Jeonbuk National University Medical School, Jeonju, Republic of Korea; ‡ Department of Poultry Science, Sylhet Agricultural University, Sylhet, Bangladesh; § The Animal Molecular Genetics & Breeding Center, Jeonbuk National University, Jeonju, Republic of Korea; # Department of Agricultural Convergence Technology, Jeonbuk National University, Jeonju, Republic of Korea

**Keywords:** broiler, heat stress, growth performance, gluconeogenesis, immunity

## Abstract

This study determined the relationship between inflammation and gluconeogenesis level in broilers in different durations of heat stress. A total of 240 Ross 308 broilers were offered control and heat stress temperature from 21 to 35 d post-hatch, each experimental group had 8 replications, and each replication obtained 15 broilers. The temperature in the control (**Ctrl**) group and heat stress group were maintained at 24 ± 1°C and 34 ± 1°C, respectively throughout the experimental period. Based on the duration of heat stress, the heat stress group was divided into 2 subgroups, like, 7-d heat stress (28-day-old broiler) designated ST group and 14-d heat stress (35-day-old broiler) designated the LT group. The ad libitum commercial feed and fresh water were provided to all experimental broilers during the experiment. The growth performance of experimental broilers was calculated at 35 d. However, the liver and blood samples were collected from the Ctrl group in 21 d, as well as these samples were collected from the heat stress ST and LT groups in 28-d and 35-d, respectively. Obvious gene expression of immunity, gluconeogenesis, glycogenolysis, and glycogenesis, as well as glucose-6-phosphate dehydrogenase and adenosine triphosphate was determined in the liver sample. The blood glucose concentration and histopathology of the liver was also examined in the different grouped broilers. Body weight, weight gain, and feed intake significantly decreased in the 35-d heat stress group than the Ctrl group. However, the feed conversion ratio increased at the 35-d heat stress group than the Ctrl group. The amount of glucose-6-phosphate dehydrogenase was significantly higher in ST and LT groups than Ctrl, whereas the blood glucose level was downregulated in the LT group. The amount of adenosine triphosphate was significantly decreased in the LT group than the Ctrl and ST groups. Heat stress acts as an impediment to the general relation between gluconeogenesis and immunity, as well as changes cellular structure. This experiment contributed to the establishment of a relationship between gluconeogenesis and immunity, which affects the growth performance of broilers during heat stress.

## INTRODUCTION

Heat stress negatively affects the wellbeing and growth performance of broilers and poses a challenge to proper metabolism and immunity. Generally, glucose metabolism decreases during heat stress for maintaining body temperature by restricting feed intake (Syafwan et al., 2011). Furthermore, heat stress can alter the energy and immune status of broilers (Segerstrom et al., 2007). Broilers produce energy via glycolysis, and the liver plays a pivotal role in this process (Rui, 2014). During fasting, glucose is synthesized in the liver via gluconeogenesis (Han et al., 2016), instead of glycolysis. In addition, the liver plays a crucial role on maintaining glucose homeostasis during prolonged starvation to meet the energy demands of the broilers (Zhang et al., 2019). In general, carbohydrates absorbed in the gastrointestinal tract are transported to the liver via the portal vein and are then converted to glucose, which subsequently serves as the metabolic fuel for the entire body. However, chronic heat stress alters liver histology, for example, the actual architecture of liver sinusoids (Chen et al., 2013; Li et al., 2014). Further, liver damage can lead to a loss of its energy-producing capacity (Nishikawa et al., 2014).

Broilers under heat stress maintain their body temperature by reducing metabolism (Conte et al., 2018), that is, reducing glycolysis, which concurrently increases gluconeogenesis (Berg et al., 2002). Consequently, broilers under heat stress exhibit low blood glucose levels (Morera et al., 2012). This decrease in glycolysis ultimately reduces the synthesis of adenosine triphosphate (**ATP**) (Yetkin-Arik et al., 2019). In addition, such disturbed glycolysis cannot maintain glucose homeostasis (Guo et al., 2012). Subsequently, glucose is produced from pyruvate and other non-carbohydrate substrates by gluconeogenesis. Interestingly, heat stress activates the gluconeogenesis process for glucose synthesis, which is followed by the transport of the synthesized glucose to various parts of the body to maintain cellular functions (Jastrebski et al., 2017). We hypothesize that the gluconeogenesis pathway influences the immune system of broilers.

The immune system plays a crucial role in maintaining the tissue stability of broilers by protecting against infectious agents (Ganeshan and Chawla, 2014) and is thus involved in environmental stress responses (Ganeshan and Chawla, 2014). Heat stress reduces the immune function of the animal through innate and humoral immune response, for instance, chronic heat stress induces the HPA axis and increase the glucocorticoids in the periphery level, that prevent the synthesis of proinflammatory cytokines (Bagath et al., 2019). The immune system is further influenced by the glucose stored in broiler cells (Von Ah Morano et al., 2020). Pro- and anti-inflammatory cytokines regulate cellular metabolism; for example, certain cytokines positively or negatively affect metabolic function (Shi et al., 2019). Moreover, there is a close relationship between the immune system and metabolism (Donath and Shoelson, 2011). It has been reported that metabolism is influenced by proinflammatory cytokines including tumor necrosis factor alpha (***TNF.α***), interleukin (***IL***) *1β*, and *IL6* by troubling the insulin and lipid signaling pathways (Dinarello et al., 2009; Shi et al., 2019). Furthermore, anti-inflammatory cytokines reduce the glucose concentration in cells (Wang and Ye, 2015), and conjugated cytokines reduce hepatic glucose concentration in blood. A previous study reported that *IL1β* and *IL10* reduce liver gluconeogenesis by 52% (Yerkovich et al., 2004).

Here, we demonstrate that chronic heat stress promotes gluconeogenesis and negatively affects the immune status in broilers. To the best of our knowledge, this is the first report on the relationship between gluconeogenesis and the liver immune status of broilers subjected to different durations of chronic heat stress. This study aimed to investigate the relationship between gluconeogenesis and inflammatory cytokines in 2 different durations of heat stress.

## MATERIALS AND METHODS

### Ethics Statement

All experiments were performed following the relevant guidelines and regulations outlined by Jeonbuk National University. The experimental procedure was approved by the Animal Experiment Administration Committee of Jeonbuk National University (approval number: CBNU2018-097). All work was undertaken ensuring minimal distress to broilers throughout the experiments.

### Broiler Management and Experimental Design

A total of 240 1-day-old Ross-308 broiler chicks were obtained from a local hatchery. The broilers were raised in cages (length × width × height, 190 cm × 120 cm × 50 cm) in an environmentally controlled farm house. The farm temperature was maintained at 33 ± 1°C during d 1–3, 30 ± 1°C during d 4 to 7, and 27 ± 1°C during days in 8 to 21. The experimental broilers were randomly divided into the heat stress and control (**Ctrl**) groups on d 20. Each group consisted of 8 replicates with 15 birds per replicate, resulting in a total of 120 broilers per treatment group. The Ctrl and heat stress groups were maintained at 24 ± 1°C and 34 ± 1°C, respectively, from d 21 to 35. The relative humidity was maintained at approximately 50% for both the Ctrl and heat treatment groups. The heat stress group was further divided into 2 subgroups according to the duration of the heat treatment. Birds in one group were subjected to heat stress for 7 d (d 22–28), which we have designated as the ST group, whereas those in the other group were subjected to heat stress for 14 d (d 22–35), which we have designated as the LT group (Figure S1). The birds were maintained under continuous light (20 h) with 4 h dark period during the experiment (22–35 d). The feed (Table S1) and water were provided ad libitum throughout the experimental period.

### Sample Collection

The body weight was measured on d 21, indicating the initial body weight, and then again on d 35, along with the feed intake of broilers in each replicate, to calculate the weight gain and feed conversion rate (**FCR**). A total of 8 broilers were randomly selected from each group for sample collection. Blood samples were collected from the wing vein and the birds were then immediately sacrificed by cervical dislocation. Liver tissue samples were collected and washed with PBS saline, immediately snap-frozen with liquid nitrogen, and stored at −80°C until further analysis. Three different broiler's fresh liver tissue samples from each group were preserved in 4% paraformaldehyde for histopathological analysis. Serum was separated from blood by centrifugation at 3,000 × *g* for 15 min at 4°C, and was then stored at −80°C until further analysis.

### Growth Performance

The body weight gain and FCR of broilers were calculated on d 35 from the total body weight and feed intake as follows:

Bodyweightgain=Finalbodyweight(day35)−Initialbodyweight (day 21)

$$\text{FCR}=\frac{\text{Total}\;\text{feed}\;\text{intake}\;\left(\text{day}\;21-\text{day}\;35\right)}{\text{Total}\;\text{body}\;\text{weight}\;\left(\text{day}\;21-\text{day}\;35\right)}$$

### Measurement of Glucose Level in Blood

The effect of different durations of chronic heat stress on the blood glucose levels was evaluated. Briefly, glucose was determined from the collected serum using a commercial colorimetric glucose test kit (Asan Pharmaceutical Co. Ltd., Seoul, Republic of Korea), following the manufacturer's instructions, and absorbance was measured at 500 nm using a spectrophotometer (Multiskan GO Microplate Spectrophotometer, Thermo Scientific, MA).

### Measurement of G6PDH Activity

The G6PDH activity was determined using a colorimetric assay kit (Cell Biolabs, Inc., San Diego, CA), according to the manufacturer's instructions. The assay kit included a G6PDH enzyme and had a detection sensitivity limit of ~1 mU/mL. Liver tissue (100 mg) was homogenized in 1 mL cold lysis buffer by using a homogenizer (IKA, T10 basic, Ultra-Turrax, Seoul, Republic of Korea). The sample and reagent were mixed in a 96-well plate and the reaction was incubated at 37°C for 15 min. The absorbance of each sample was recorded at 450 nm using a spectrophotometer (Multiskan GO Microplate Spectrophotometer, Thermo Scientific, MA).

### ATP Quantification

The ATP concentration was analyzed using a colorimetric ATP assay kit (Abnova, Walnut, CA), following the manufacturer's instructions. This assay kit can detect down to 50 pmol (1 μM) of ATP. Liver tissue (10 mg) was homogenized in 100 μL ATP assay buffer by a using homogenizer (IKA, T10 basic, Ultra-Turrax, Seoul, Republic of Korea). The sample and reagents were mixed and the reaction was incubated at 25°C for 30 min. The absorbance of each sample was determined at 570 nm using a spectrophotometer (Multiskan GO Microplate Spectrophotometer, Thermo Scientific, MA).

### Gene Analysis

Total RNA was extracted from frozen broiler liver samples using TRIzol reagent (Invitrogen, New York, NY), according to the manufacturer's instructions, and quantified using a Nanodrop spectrometer (Thermo Scientific, MA) based on the absorbance at 230 nm and 260 nm/280 nm. The RNA was then reverse-transcribed into cDNA using the iScript cDNA synthesis kit (Bio-Rad, CA), according to the manufacturer's instructions. Quantitative real-time PCR was performed on a CFX96TM Real-Time PCR detection system (Bio-Rad, CA), using the SYBR master mix (Toyobo, NY) and with the following cycling conditions: 95°C for 30 s; 40 cycles of 95°C for 5 s and 58°C for 5 s; melting temperature of 65°C to 95°C for 5 s. All targeted primers used in the study are listed in Table 1. All samples were tested using 8 replicates. The mRNA expression level of genes of interest was quantified relative to that of glyceraldehyde-3-phosphate dehydrogenase by the 2^−ΔΔCt^ method (Livak and Schmittgen, 2001). The PCR primer pair efficiency (E) was calculated from the slope of the standard curve, using the equation E= [10(−1/slope) − 1] × 100% (Radonić et al., 2004) (Table 1). The efficiency of the targeted primer pair should be 90 to 110%.

**Table 1:** Primer sequences, amplicon characteristics, efficiency of different gene for RT-qPCR analysis.

Gene symbol	Accession number	Primer sequences (5′−3′)	Amplicon size (bp)	TM (°C)	Efficiency (%)
PC	NM 204346.1	F: AAGACGCTGCACATCAAAGC	113	60	104.13
		R: TGGGTGTCTCTCACGAGGAT			
PEPCK	NM 205471.1	F: GGCAATCAATCCAGAAAACG	139	56	109.33
		R: CCAATAGACACCCCCATCAC			
FBP	NM 001278048.1	F: GAATAGCAACAACAGGGCATC	81	58	106.01
		R: ATCCCAAGACAACAGGCACT			
GYS1	XM 025145463.1	F: CACGCACCAACAACTTCAAC	122	58	108.23
		R: CACCAGCAGCGACTCATAGA			
PYG	NM 204392.1	F: CTTTGGGATGAGGGTGGAG	105	58	109.17
		R: ATCTGGTCAACTGCCTGCTT			
IL 1	XM 015297469.1	F:GCATCAAGGGCTACAAGCTC	131	59	100.19
		R:CAGGCGGTAGAAGATGAAGC			
IL 2	XM 015276098.2	F: ACCGGAAGTGAATGCAAGAT	212	58	106.53
		R: AGTGGTCCCAGAATGGACAG			
IL 4	NM 001007079.1	F: GCTCTCAGTGCCGCTGATG	61	60	105.63
		R: GGAAACCTCTCCCTGGATGTC			
IL 6	XM 015281283.2	F: CTCCTCGCCAATCTGAAGTC	100	59	105.64
		R: CCCTCACGGTCTTCTCCATA			
IL 10	XM 025143715.1	F: CTGAAGGCGACGATGC	406	55	101.43
		R:TTCCTCCTCCTCATCAGC			
TNF.α	XM 015294125.2	F:AGGCCAGATGGGAAGGGAATGAA	219	63	104.14
		R:GAAGAGGCCACCACACGACAG			
GAPDH	NM 204305.1	F:CACCCTCAAGATTGTCAGC	98	60	109.32
		R:TAAGTCCCTCCACGATGC			

Abbreviations: *FBP*, fructose-1,6-bisphosphatase; *GYS1*, liver glycogen synthase; *GAPDH*, glyceraldehyde-3-phosphate dehydrogenase; *IL-1*, Interleukin 1; *IL2*, Interleukin 2; *IL4*, Interleukin 4; *IL6*, Interleukin 6; *IL10*, Interleukin 10; *PC*, pyruvate carboxylase; *PEPCK*, phosphoenolpyruvate carboxykinase; *PYG*, glycogen phosphorylase, *TNF.α*, tumor necrosis factor α.

### Histopathology

Liver tissue samples were fixed in 4% paraformaldehyde (Sigma-Aldrich, Saint Quentin Fallavier, France) immediately after collection. All samples were embedded in paraffin using an auto processor (Excelsior ES, Thermo Scientific, MA), and 5-µm-thick sections were prepared and stained with hematoxylin and eosin (H&E) (Qiu et al., 2016). Histopathological changes in the liver tissue were observed using a Leica DM2500 microscope (Leica Microsystems, Wetzlar, Germany) at 100 × magnification.

### Statistical Analyses

Data are expressed as mean ± standard error of the 8 replicates in each group. Glucose, glucose-6-phosphate dehydrogenase (**G6PDH**), ATP, data were subjected to analysis of variance (**ANOVA**) followed by Duncan's multiple range test. Growth performance and different genes expression were analyzed by Student's *t* test, and the former was further subjected to ANOVA. Principal component analysis (**PCA**) was performed using SAS version 9.4 (SAS Institute Inc., Cary, NC). A heat map was developed to cluster genes based on their correlations. The gene-gene interaction network was constructed including gluconeogenesis and immunity-related genes. Statistical significance was set at *P* < 0.05, *P* < 0.01, and *P* < 0.001.

## RESULTS

### Chronic Heat Stress Alters Metabolite Levels

**Figure 1:**
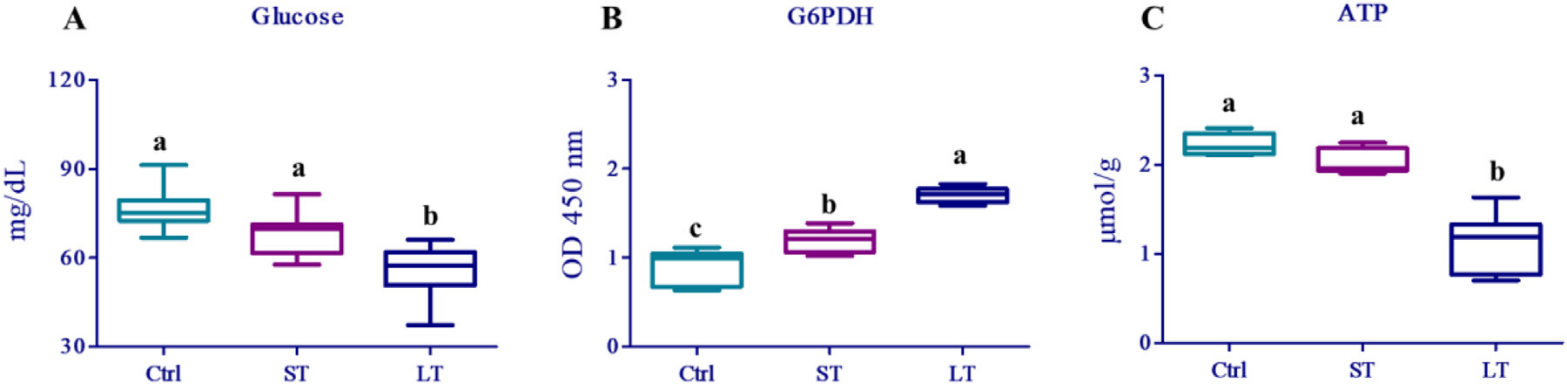
Chronic heat stress has an effect on glucose, ATP levels, and G6PDH activity. (A) Blood glucose level was decreased in the LT group. (B) G6PDH activity was higher in the liver of broilers in the ST and LT groups under heat stress than in that of Ctrl group. (C) The ATP level was remarkably lower in the liver of broilers in the LT group under chronic heat stress than in that of Ctrl and ST groups. ^a-c^ Values with different superscripts within the same column are significantly different (*P* < 0.05). Abbreviations: ATP, adenosine triphosphate; Ctrl, Control; G6PDH, glucose-6-phosphate dehydrogenase.

We examined the blood glucose and liver ATP levels and the activity of G6PDH (Figure 1). The blood glucose levels of broilers subjected to heat stress for different durations are presented in Figure 1A. The glucose concentration was significantly (*P* < 0.05) lower in the 14 days’ heat-stressed (**LT**) group than in the Ctrl and 7 days’ heat-stressed (**ST**) groups, whereas the glucose concentration of the Ctrl and ST groups was not significantly different.

The effect of exposure to different durations of chronic heat stress on liver G6PDH activity is presented in Figure 1B. The G6PDH activity was significantly (*P* < 0.05) higher in the LT group than in the Ctrl and ST groups. Further, the G6PDH activity in the ST group was significantly (*P* < 0.05) higher than that in the Ctrl group.

The effect of exposure to different durations of chronic heat stress on liver ATP production is shown in Figure 1C. The ATP production was significantly lower (*P* < 0.05) in the LT group than in the Ctrl and ST groups.

### Immunity- and Gluconeogenesis-Related Gene Expression in Liver

**Figure 2:**
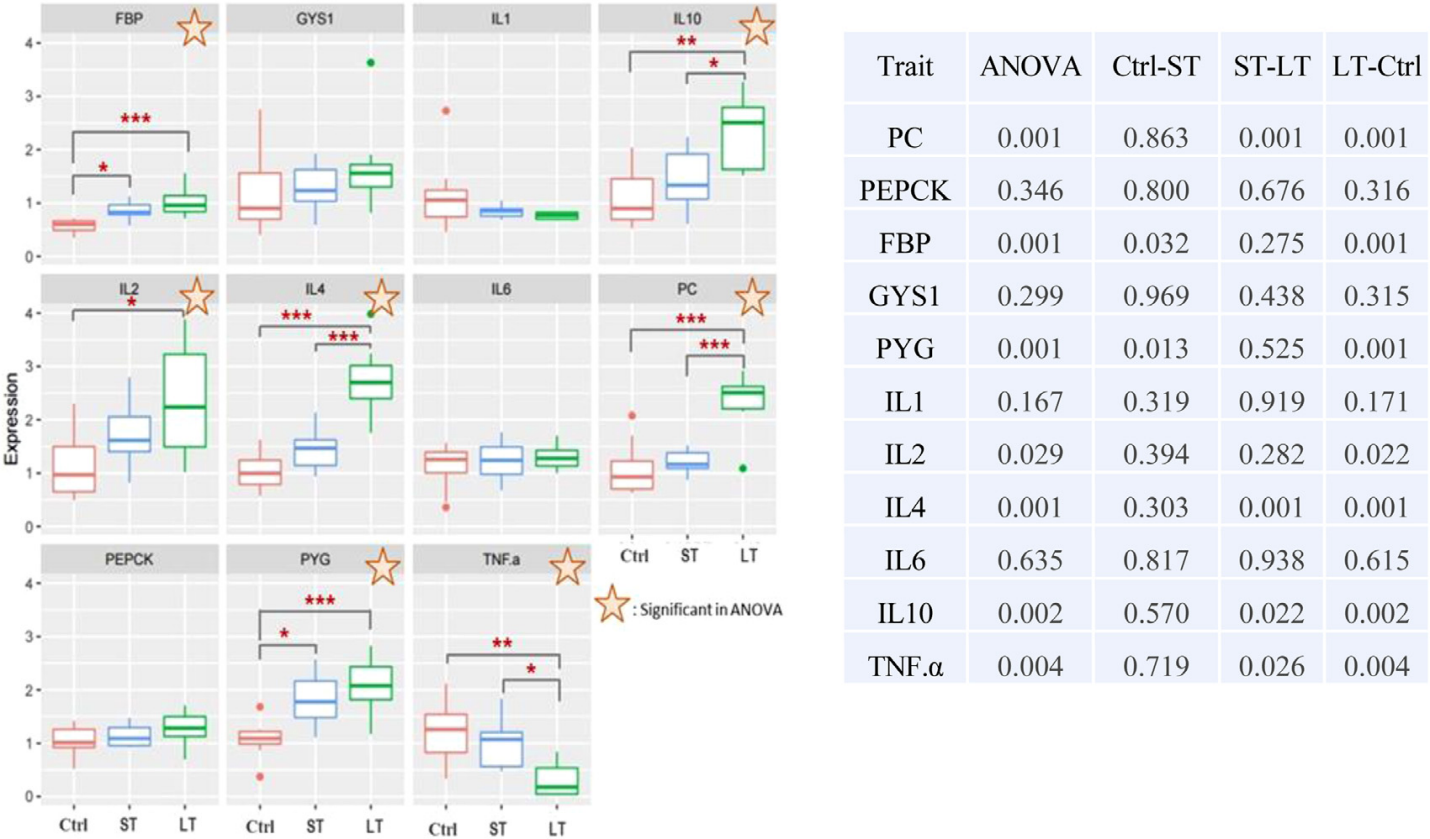
Expression of immunity- and gluconeogenesis-related genes. The box plot represents the relative expression levels of different immunity- and gluconeogenesis-related genes. The star symbol indicates a significant difference between the treatment and Ctrl groups. The table presents the *P*-value of ANOVA between two groups. Asterisks indicate significantly different results in the *t* test (*P < 0.05, **P < 0.01, ***P < 0.001).

The effect of exposure to different durations of chronic heat stress on immunity and gluconeogenesis-related gene expression is shown in Figure 2. There was no significant difference could be detected in the expression of phosphoenolpyruvate carboxykinase (***PEPCK***) and glycogen synthase (***GYS1***) genes in the liver among any of the treatment groups. The expression level of the pyruvate carboxylase (***PC***) gene was significantly (*P* < 0.001) higher in the LT group than in the Ctrl and ST groups. Similarly, the expression level of the fructose-1,6-bisphosphatase (***FBP***) gene was significantly (*P* < 0.001) higher in the LT group than in the Ctrl. Among the latter groups, *FBP* expression was significantly (*P* < 0.05) higher in the ST group than in the Ctrl group. The expression level of the glycogen phosphorylase (***PYG***) gene was significantly higher in the ST and LT groups than in the Ctrl group. There was no significant difference was detected in the expression of *IL1* and *IL6* in the liver among any of the treatment groups. The *IL2* expression level in the liver was significantly (*P* < 0.05) higher in the LT group than in the Ctrl group. The expression level of *IL4* and *IL10* was significantly higher in the LT group than in the Ctrl and ST groups, whereas that of *TNF.α* was significantly lower in the LT group than in the Ctrl and ST groups.

### Correlation Between Gluconeogenesis and Immunity Genes

**Figure 3:**
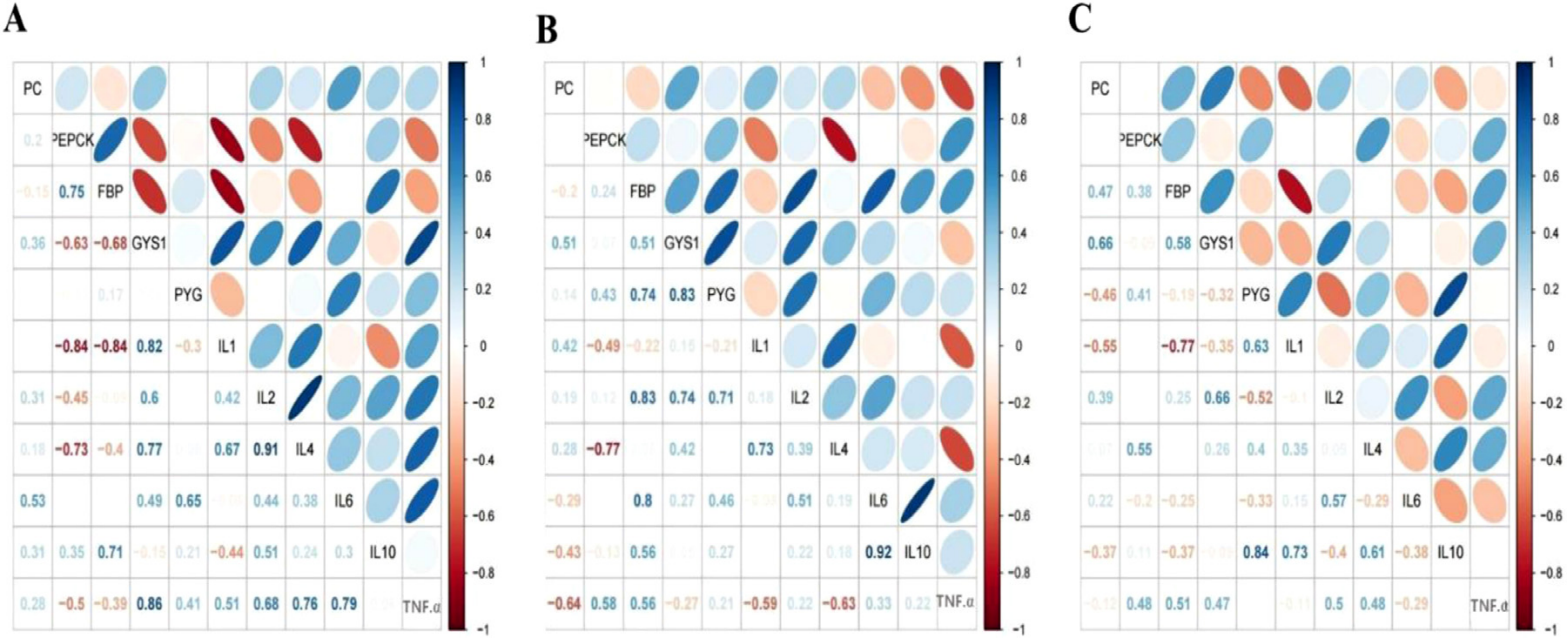
Heat map demonstrating the correlation among various gluconeogenesis and immunity parameters. The correlation was calculated for each gluconeogenesis and immunity parameter and is indicated in each cell of the matrix. Blue and brown colors indicate positive and negative correlations, respectively. (A) Correlation among different immunity and gluconeogenesis parameters in the Ctrl group. (B) Correlation among different immunity and gluconeogenesis parameters in the ST group. (C) Correlation among different immunity and gluconeogenesis parameters in the LT group.

Correlations between the expression of 11 genes (*PC, PEPCK, FBP, GYS1, PYG, IL1, IL2, IL4, IL6, IL10*, and *TNF.α*) were assessed in the liver of broilers from the 3 treatment groups (Figure 3). In the Ctrl group, the *PEPCK* was significantly co-related with *FBP, IL1*, and *IL4; FBP* was significantly correlated with *IL1; GYS1* was significantly correlated with *IL1, IL4*, and *TNF.α; IL2* was correlated with *IL4* (Figure 3A). In the ST group, the *FBP* was correlated with *PYG, IL2*, and *IL6; GYS1* was correlated with *PYG* and *IL2; PYG* exhibited a strong correlation with *IL2; IL4* was correlated with *PEPCK* and *IL1; IL6* was strongly correlated with *IL10* (Figure 3B). In the LT group, *FBP* was correlated with *IL1*, and *IL10* was correlated with *PYG* and *IL1* (Figure 3C).

### Comparison Using Correlation-Based Clustering

**Figure 4:**
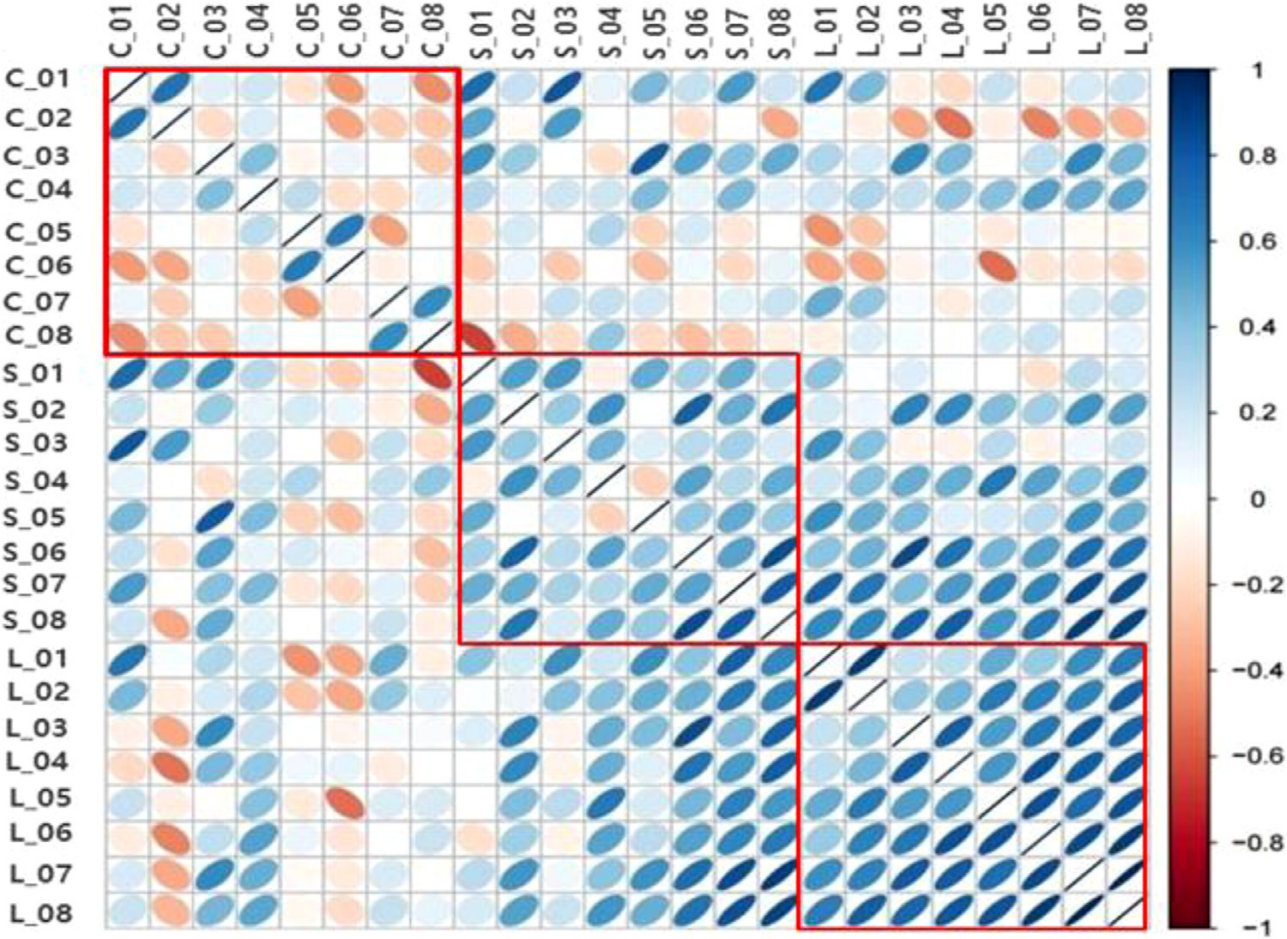
Hierarchical clustering demonstrating distinct immunity and gluconeogenesis-related metabolite profiles of the Ctrl (C), ST (S), and LT (L) groups. Blue and brown colors indicate higher and lower gene expression levels, respectively. The heat map presents the expression of different genes in a grouped form.

The differences in the correlation of the tested genes among the Ctrl, ST, and LT groups are illustrated in Figure 4. Our result detects correlation using a grouped cluster, where merges 11 genes in each group. The cluster demonstrated the correlation among Ctrl, ST, and LT Genes encoding cytokines, which are involved in the progression of the inflammatory response, and gluconeogenesis-related genes clustered together. These clusters provide evidence of the correlation between different genes in the three groups. All genes appeared to be positively correlated in the LT group, whereas those in the Ctrl group were negatively correlated. Notably, some genes in the ST group were only moderately correlated.

### PCA

We employed PCA to compare the treatment groups considering the different durations of heat stress exposure. Associations among the tested parameters were compared across the Ctrl, ST, and LT groups. The first 2 principal components were selected as they explained 71.94% of the variance among the 11 parameters in the 8 samples analyzed for each group (PC1 = 54.93% and PC2 = 17.01%). We noted that the expression of all genes was different between that in the Ctrl, ST, and LT groups (Figure S3), whereas it was considerably different from that in the Ctrl group.

**Figure 5:**
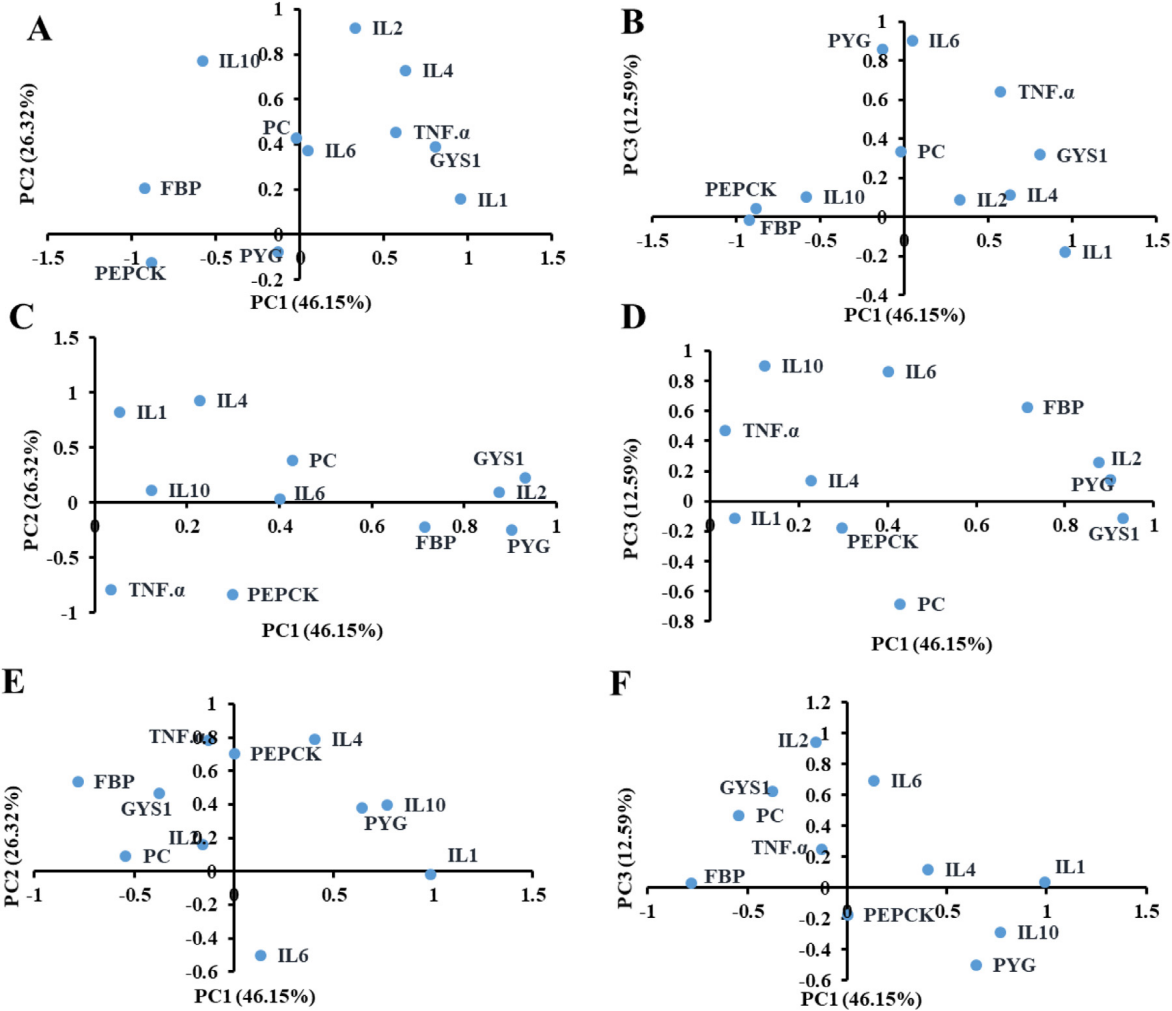
Comparison of the association between various parameters based on principal component analysis (PCA). PCA results of the Ctrl (A, B), ST (C, D), and LT (E, F) groups are indicated. PC1, PC2, and PC3 were selected as they explained the most variation.

We further performed PCA on the 11 parameters to determine the association among the 4 gene groups, that is, glycogenolysis, glycogenesis, gluconeogenesis, and immunity (Figure 5). Association among the parameters was compared across the Ctrl, ST, and LT groups. The first 3 principal components explained 85.06% of the variance among the 11 analyzed parameters (PC1 = 46.15%, PC2 = 26.32%, and PC3 = 12.59%). In the Ctrl group, the gluconeogenesis-related parameters *FBP* and *PEPCK* were closely associated (Figure 5B). Furthermore, the immunity parameter *IL1* was negatively and positively associated with gluconeogenesis (*PEPCK* and *FBP*), and *GYS1* parameters, respectively. The immunity parameter *IL4* was negatively correlated with *PEPCK* and positively correlated with *GYS1* and *IL2. TNF.α* and *GYS1* were present in the region of positive PC1 and PC2 loadings. The associations between glucose synthesis and immunity were altered in the ST group due to exposure to heat stress for 7 d (Figure 5C and 5D). The immunity parameter IL2 was positively associated with *FBP, PYG*, and *GYS1*. The *PEPCK* was negatively associated with *IL4*, whereas it was positively correlated with *IL1* (Figure 5C). The gluconeogenesis parameter *FBP* and immunity-related parameters *IL6* and *IL10* were placed in the same quadrant, indicating a positive correlation (Figure 5D). In the LT group, the glucose synthesis and immunity genes were distributed in different quadrants with positive and negative associations, as indicated by PC1, PC2, and PC3 loadings, which was likely influenced by the exposure to heat stress for 14 d (Figure 5E and 5F). The immunity parameter *IL1* was negatively correlated with *FBP* (Figure 5E), whereas *IL1, IL10*, and *PYG* were positively correlated (Figure 5F). The principal component loadings for the different variables in the Ctrl, ST, and LT groups are listed in Tables S2, S3, and S4, respectively.

### Gene-Gene Interaction Analysis

**Figure 6:**
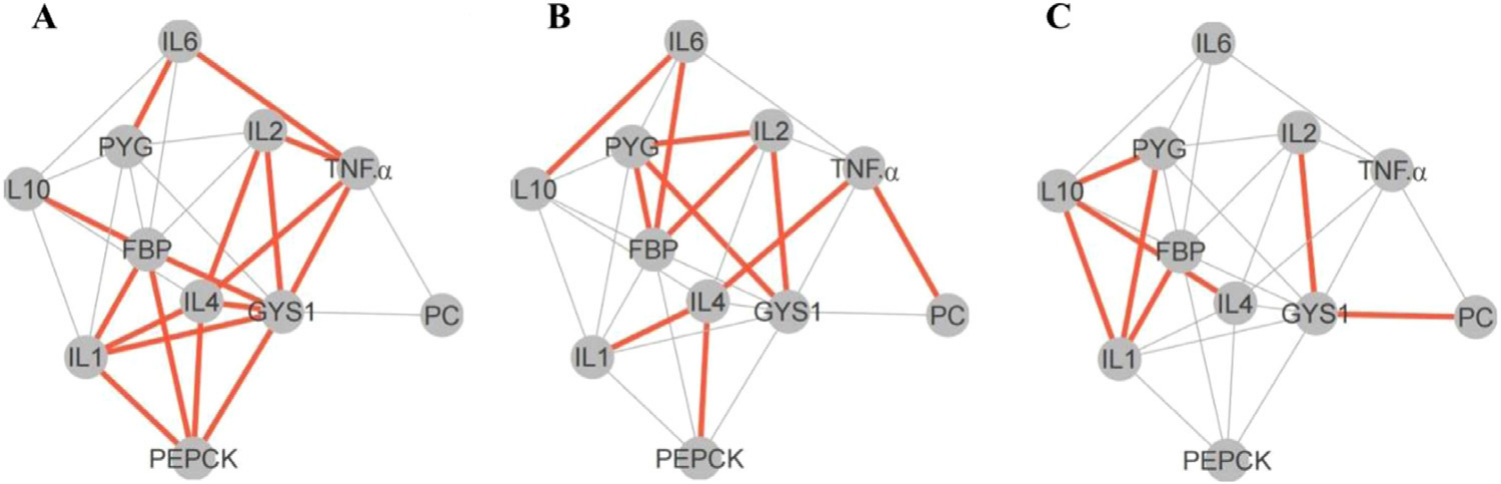
Gene interaction networks indicating inhibition of gene-gene interaction on heat exposure. The direct gene-gene interactions are indicated by lines, with prominent brown and gray lines indicating strong and weak interactions, respectively. (A) Gene-gene interaction in the Ctrl group. (B) Gene-gene interaction in the ST group. (C) Gene-gene interaction in the LT group.

The gene-gene interactions of gluconeogenesis and immunity related genes under chronic heat stress are presented as 3 networks (Figure 6), corresponding to the Ctrl, ST, and LT groups and each including the 11 tested genes. In the first network, that is, the Ctrl group (Figure 6A), most genes were strongly correlated with each other. The *GYS1* was strongly correlated with *PEPCK, FBP, IL1, IL2, IL4*, and *TNF.α*. However, *PC* was not strongly correlated with other genes in the Ctrl group. The second network indicates the effect of 7 days’ heat stress (ST group) on gluconeogenesis- and immunity-related gene-gene interaction (Figure 6B). Here, the *IL4* was remarkably correlated with *PEPCK, IL1*, and *TNF.α; FBP* was strongly correlated with *PYG, IL2*, and *IL6; PYG* was correlated with *FBP, GYS1*, and *IL2*. Interestingly, *PC* interacted with *TNF.α* in this group. Finally, in the third network (Figure 6C), that is, the LT group, *IL1* and *IL10* were strongly correlated with each other and with *PYG* and *FBP*.

### Construction of Gene Expression Networks

**Figure 7:**
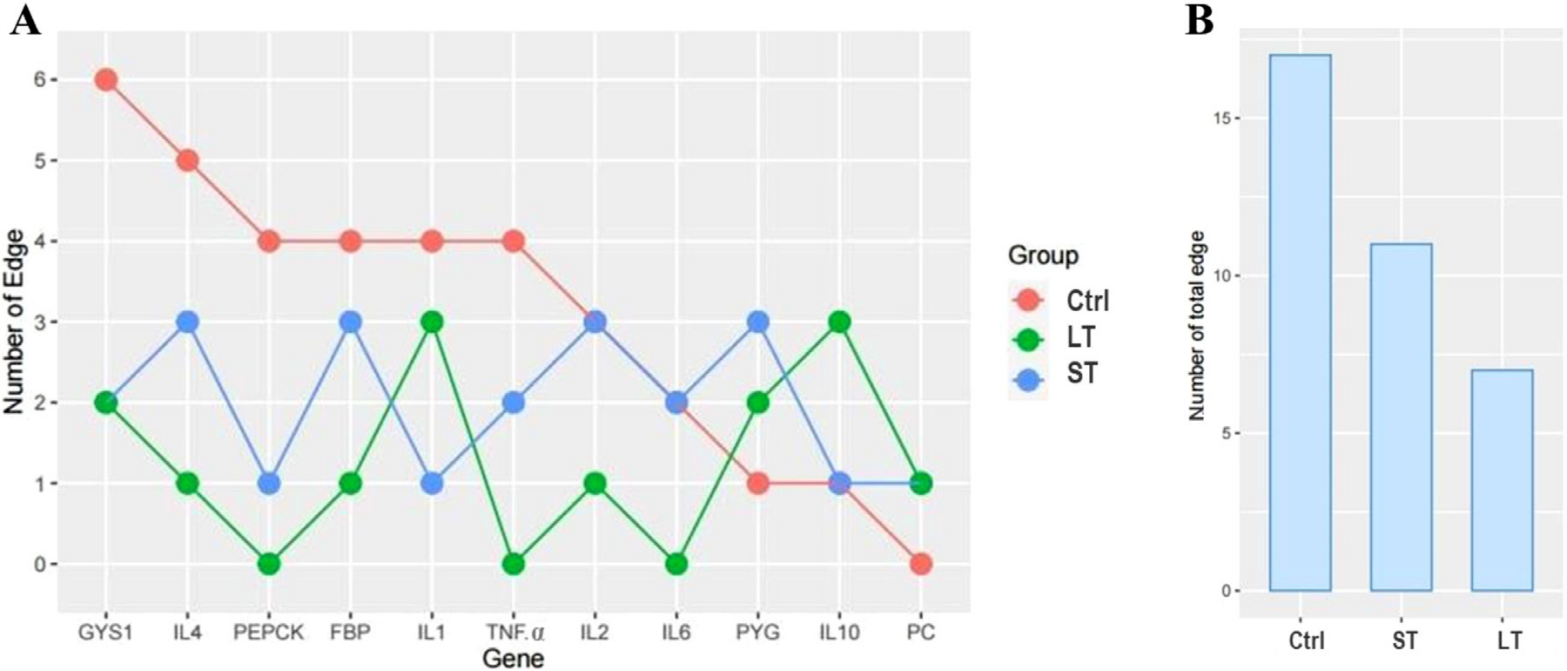
Summary of the gene expression network for different gluconeogenesis and immunity parameters. Distribution of the edge number in the expression network of gluconeogenesis and immunity genes under different durations of heat stress. (A) Different genes exhibiting graphically diverse edge numbers in different groups. (B) The summary of total edge number in different groups.

We focused on the differences in gene expression level, that is, lower or higher, in the gene expression networks of the 3 treatment groups. The frequency distribution of the number of edges is shown in Figure 7A. A higher number of edges was noted in the Ctrl group, whereas fewer edges were detected in the LT group (Figure 7B). The ST group presented a fewer number of edges. This indicates that the core gene of the Ctrl group was strongly correlated with a higher number of genes, whereas lower number of genes were correlated with each other in the LT group.

### Growth Performance of Broilers

The precondition for satisfactory broiler growth performance is favorable environmental temperature. Therefore, we examined the effect of chronic heat stress on broiler growth performance by measuring the final body weight and feed intake of 35-day-old broilers. The effects of chronic heat stress on broiler growth performance are presented in Table 2. The final body weight of broilers was significantly lower in the heat-stressed groups than in the Ctrl group on d 35. Similarly, the feed intake and body weight gain were significantly lower in the heat-stressed group than in the Ctrl group on d 35. Conversely, the FCR in the heat-stressed group was significantly higher when compared with that in the Ctrl group.

**Table 2:** Growth performance of broiler.

Item	Control	Heat stress	*P* value
Initial day (21 d)			
Body weight (g/ broiler)	1018.81 ± 7.50	1030.41 ± 7.47	0.98
35 d			
Body weight (g/ broiler)	2366.83 ± 63.24	1634.67 ± 50.90^⁎⁎^	0.01
21–35 d			
Feed intake (g/ broiler)	2296.45 ± 73.95	1536.60 ± 65.02^⁎⁎^	0.01
Weight gain (g/ broiler)	1346.34 ± 76.15	710.70 ± 66.23^⁎⁎^	0.01
FCR	1.72 ± 0.04	2.40 ± 0.17*	0.05

Effect of chronic heat stress on the growth performance of broilers (n = 8). Body weight, feed intake, weight gain, and feed conversion ratio (FCR) in control and treatment groups, All the data were presented by mean ± SE (standard error).

Asterisk (*) represents statistical difference between control and treatment groups, *P < 0.05, **P < 0.01.

### Chronic Heat Stress-Induced Liver Damage

The effect of exposure to different durations of chronic heat stress on liver damage is presented in Figure S4. The liver of broilers subjected to chronic heat stress, that is, LT and ST groups, exhibited necrotic cells, whereas no such cells were noted in the Ctrl group. However, liver damage was minimal in the ST group when compared with that in the LT group.

## DISCUSSION

Exposure to heat is the most common stressor of broilers in tropical, subtropical, and even milder climatic regions. Poor growth and death of broilers are pivotal factors limiting the development of animal husbandry in hot regions. Newly-hatched broiler chicks cannot regulate their own body temperature due to their poorly developed thermoregulatory systems. Therefore, artificial brooding with regulated temperature is required for survival and organ development (Akşit et al., 2010). However, high temperatures reduce the growth performance of animals by inducing hepatocyte necrosis and lowering metabolism and energy production (Thompson et al., 2014; Ma et al., 2019).

**Figure 8:**
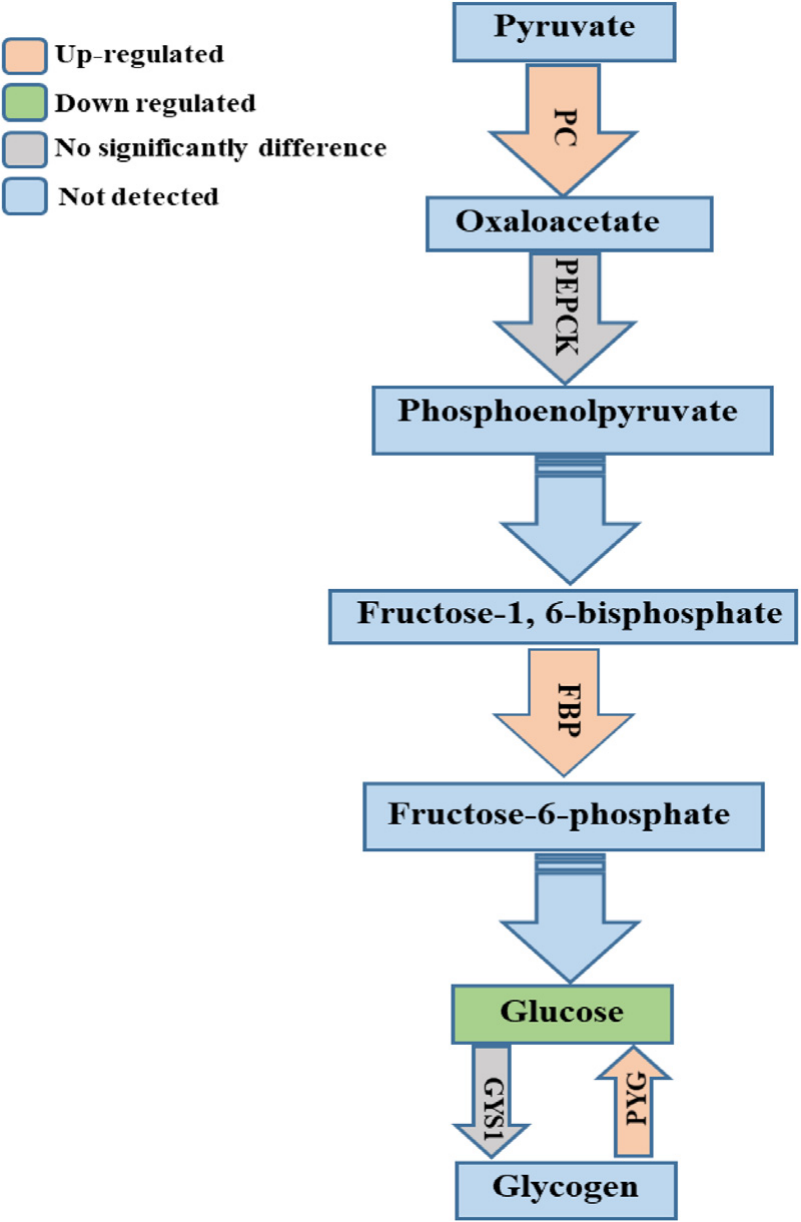
Schematic design of the gluconeogenesis pathway. Illustration of glycogenesis, glycogenosis, and gluconeogenesis pathways with integrated metabolomic and transcriptomic data from broiler liver samples under chronic heat stress. The rectangles indicate metabolites, unbroken arrows indicate genes, and broken arrows indicate skipped steps. The pathway indicates if a gene was detected and if it was up- or downregulated or remained unchanged under heat stress.

The liver plays a crucial role in metabolism, and its metabolic activity is regulated by insulin and various metabolic hormones. The nutrients present in the feed, including glucose and fatty and amino acids, are absorbed into the bloodstream in the gastrointestinal tract and transported to the liver via the portal circulation. Glucose is then converted into glycogen by glycogen synthase and stored in the liver (Agius, 2008). Conversely, during short periods of fasting, glucose is produced from the stored glycogen in the liver via glycogenolysis by glycogen phosphorylase (Rui, 2014). In addition, the amount of glycogen reduces during prolonged fasting, in which case the hepatocytes produce glucose by gluconeogenesis using pyruvate, lactate, and amino acids (Rui, 2014). In the cytosol, lactate dehydrogenase oxidizes lactate into pyruvate, which is subsequently converted into oxaloacetate by pyruvate carboxylase (Rui, 2014). Furthermore, heat stress increases PEPCK (Qu et al., 2016), which converts oxaloacetate to phosphoenolpyruvate (She et al., 2000). However, it has been reported that in the mouse model, the liver can produce glucose and maintain blood glucose levels without PEPCK during short fasting periods (Burgess et al., 2004). Phosphoenolpyruvate is converted into fructose-1,6-bisphosphate through a series of biochemical reactions, which is then converted to fructose-6-phosphate by fructose-1,6-bisphosphatase. Moreover, the fructose-1,6-bisphosphatase increase in rabbit liver under prolonged fasting (Park et al., 2020). In this study, the expression profiles of glycogenesis-, glycogenolysis-, and gluconeogenesis-related genes in broiler liver under chronic heat stress were constructed. The expression levels of glycogenolysis- and gluconeogenesis-related genes were increased under chronic heat stress conditions (Figure 8). These results are consistent with the increase in gluconeogenesis in mouse liver to supply glucose to glucose-dependent tissues as a compensatory fuel during fasting (Liu et al., 2008). Moreover, during heat stress, increases glycogenolysis and gluconeogenesis for maintaining homeostasis of liver (Jastrebski et al., 2017). Our results revealed that gluconeogenesis is increased in the liver due to chronic heat stress.

Metabolism is closely associated with immune cell function, with both exerting an influence on each other (Alwarawrah et al., 2018). Glucose improves lymphocyte survivability and function, as well as T-cell activation and proliferation (Maciver et al., 2008). Moreover, blood glucose plays a crucial role in immunity, promoting cellular immune response (Xu and Wang, 2011). Additionally, glucose homeostasis plays an important role to protect immune cells from systemic infection (Tucey et al., 2018). Moreover, reserved energy (i.e., glycogen) plays a critical role in the homeostasis of the immune system (Fantuzzi, 2005; Schäffler et al., 2007). A previous study demonstrated that animals with high energy reserves exhibit improved immune function (Houston et al., 2007). Further, optimum carbohydrate intake promotes strong immune responses (Zhou et al., 2013). However, chronic heat stress alters the normal metabolic functions in broilers. A decrease in the blood glucose level has previously been reported in response to chronic heat stress (Morera et al., 2012), which is consistent with our results. Moreover, such a physiological condition leads to gluconeogenesis to provide cells with the required energy. Van Goor et al. (2015) reported that chronic heat stress decreases the growth performance of broilers (Van Goor et al., 2015). Our results showed a decrease in body weight, weight gain, and feed intake, as well as an increase in FCR under chronic heat stress. Furthermore, endotoxin directly damages various organs, including the liver (Bouaziz et al., 2007). Our results further demonstrated greater histopathological damage (necrosis) in the LT group than in the Ctrl group.

Hepatocytes can produce 80% to 90% of the circulating innate immunity proteins (Zhou et al., 2016). Therefore, the liver is known as an immunological organ (Racanelli and Rehermann, 2006). Proinflammatory cytokines, particularly IL1 and TNF.α, play important roles in several stages of liver disease to reduce disease severity (Niederreiter and Tilg, 2018). Moreover, IL6 plays a pivotal role in liver regeneration (Schmidt-Arras and Rose-John, 2016). It has been reported that immune cells, including monocytes and neutrophils, inhibit the secretion of proinflammatory cytokines, such as *IL1* and *TNF.α*, while inducing the secretion of anti-inflammatory cytokines, such as *IL4* and IL10 (Elenkov and Chrousos, 1999; Tracey, 2002). In contrast, a previous study reported an increase in some proinflammatory cytokines, including *IL2* and *IL6*, under chronic stress conditions (Tian et al., 2014). Our results showed that chronic heat stress increases inflammation in the liver. Moreover, chronic heat stress increases the inflammatory signaling in pig skeletal muscle and broiler small intestine (Ganesan et al., 2016; Siddiqui et al., 2020).

## CONCLUSIONS

Chronic heat stress induced marked changes in the thermophysiological traits of broilers, including liver structure, feed intake, and body weight gain, and increased FCR. Low feed intake resulted in low blood glucose levels in broilers under chronic heat stress. The decline in blood glucose levels induced gluconeogenesis in the liver, further resulting in reduced ATP production. The low cellular energy levels further influenced broiler immunity. Collectively, chronic heat stress negatively affected ATP synthesis and immunity, which may lead to poor growth performance of stressed broilers.

## Appendix Supplementary materials

Image, application 1
