# Supplementary material for: Altered relationship between gluconeogenesis and immunity in broilers exposed to heat stress for different durations
Source: Poult Sci. 2021 May 21;100(8):101274. doi: 10.1016/j.psj.2021.101274 (PMC8267598; doi:10.1016/j.psj.2021.101274)
Supplement: Supplementary file 1 [file mmc1.docx]

**Supplementary Information**

**Altered relationship between gluconeogenesis and immunity in broilers exposed to heat stress for different durations**


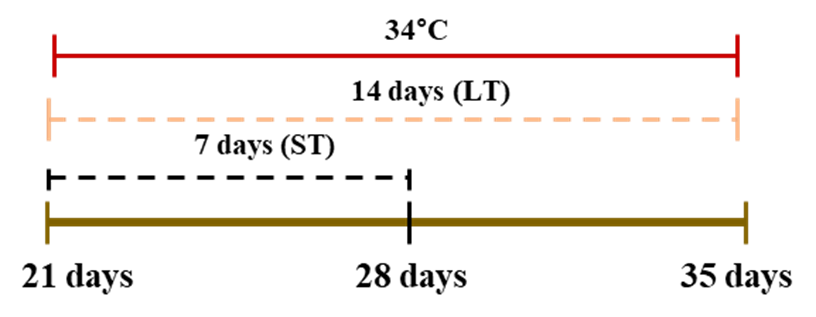


**Figure S1.** **Experimental design**. This experiment categorized three groups. The control (Ctrl) group indicates 21 days aged broiler without heat stress. The experimental groups are 7 days’ heat stress and 14 days’ heat stress exposure and samples were collected gradually at 28 and 35 days aged broiler. These two experimental groups are presented by ST and LT respectively.





**Figure S3. Principal component analysis (PCA) demonstrating profile of different experimental groups.** The PCA showing group of gene expression profiles among different time points of heat stress. We choose PC1 (54.93%) and PC2 (17.01%) for it showing good result than others. The red color indicates Ctrl, the blue color indicates ST, and the green color indicate LT group.





**Figure S4.** **Histopathological analysis.** Effect of different duration of chronic heat stress on the morphology of the cellular structure of the liver of broilers.

**Table S1.** Chemical composition of broiler feed.

| **Feed’s chemical Composition** | **Starter*** | **Finisher**** |
| --- | --- | --- |
| Crude protein | 20.0% | 19.0% |
| Crude fat | 4.0% | 4.0% |
| Calcium | 0.75% | 0.75% |
| Phosphate | 0.70% | 0.70% |
| Crude fiber | 6.0% | 5.5% |
| Crude ash | 8.0% | 8.0% |
| Met + Cys + MHA^#^ | 0.75% | 0.65% |
| ME^##^ | 3.00 Mcal/kg | 3.05 Mcal/kg |

*Starter, 0–20 day; ** Finisher, 21–35 day; ^#^Met + Cys + MHA, DL-Methionine + Cysteine + DL-Methionine hydroxyl analogue; ^##^ ME, metabolizable energy.

**Table S2.** Loadings of PC1, PC2, and PC3 of Ctrl group.

| **Variable** | **PC1** | **PC2** | **PC3** |
| --- | --- | --- | --- |
| **PC** | -0.02201 | 0.42958 | 0.33674 |
| **PEPCK** | -0.8813 | -0.12584 | 0.04242 |
| **FBP** | -0.92408 | 0.2034 | -0.01516 |
| **GYS1** | 0.80607 | 0.38826 | 0.31738 |
| **PYG** | -0.13034 | -0.07531 | 0.85837 |
| **IL1** | 0.95682 | 0.15607 | -0.1777 |
| **IL2** | 0.33038 | 0.91784 | 0.08815 |
| **IL4** | 0.62858 | 0.72948 | 0.11451 |
| **IL6** | 0.04794 | 0.37237 | 0.90357 |
| **IL10** | -0.58074 | 0.76862 | 0.10446 |
| **TNF.α** | 0.57082 | 0.45249 | 0.63924 |

PC= Principal component

**Table S3.** Loadings of PC1, PC2, and PC3 of ST group.

| **Variable** | **PC1** | **PC2** | **PC3** |
| --- | --- | --- | --- |
| **PC** | 0.42813 | 0.38272 | -0.68242 |
| **PEPCK** | 0.29777 | -0.83886 | -0.17829 |
| **FBP** | 0.715 | -0.22575 | 0.62592 |
| **GYS1** | 0.93155 | 0.2261 | -0.11628 |
| **PYG** | 0.90189 | -0.24446 | 0.14122 |
| **IL1** | 0.05479 | 0.81965 | -0.11645 |
| **IL2** | 0.87608 | 0.09626 | 0.25715 |
| **IL4** | 0.22799 | 0.93082 | 0.13712 |
| **IL6** | 0.40165 | 0.03314 | 0.86398 |
| **IL10** | 0.1238 | 0.11565 | 0.90236 |
| **TNF.α** | 0.03465 | -0.79365 | 0.46786 |

PC= Principal component

**Table S4.** Loadings of PC1, PC2, and PC3 of LT group.

| **Variable** | **PC1** | **PC2** | **PC3** |
| --- | --- | --- | --- |
| **PC** | -0.54278 | 0.09228 | 0.46811 |
| **PEPCK** | 0.00413 | 0.70034 | -0.1758 |
| **FBP** | -0.78017 | 0.53327 | 0.03322 |
| **GYS1** | -0.37281 | 0.46472 | 0.62084 |
| **PYG** | 0.64512 | 0.37815 | -0.49813 |
| **IL1** | 0.98903 | -0.0167 | 0.03482 |
| **IL2** | -0.15767 | 0.15931 | 0.94302 |
| **IL4** | 0.40593 | 0.78946 | 0.11593 |
| **IL6** | 0.13467 | -0.50379 | 0.69516 |
| **IL10** | 0.76829 | 0.39733 | -0.28657 |
| **TNF.α** | -0.12843 | 0.7819 | 0.24667 |

PC= Principal component
